# Autonomic nervous system responses in the intermediate band to cranial cutaneous stimulation

**DOI:** 10.14814/phy2.15891

**Published:** 2024-01-01

**Authors:** Micha Keller, Holger Pelz, Gero Müller, Stefan Borik, Klaus Mathiak, Johannes Mayer, Ines Repik, Armin Geilgens, Volker Perlitz

**Affiliations:** ^1^ Department of Psychiatry, Psychotherapy and Psychosomatics Medical School RWTH Aachen University Aachen Germany; ^2^ Deutsche Gesellschaft für Osteopathische Medizin e.V Buxtehude Germany; ^3^ Simplana GmbH Aachen Germany; ^4^ Department of Electromagnetic and Biomedical Engineering, Faculty of Electrical Engineering and Information Technology University of Zilina Zilina Slovakia; ^5^ JARA‐Brain, Research Center Jülich Jülich Germany; ^6^ Deutsche Gesellschaft für Osteopathische Medizin e.V Augsburg Germany; ^7^ Deutsche Gesellschaft für Osteopathische Medizin e.V Mannheim Germany

**Keywords:** 0.15 Hz rhythm band, autonomic nervous system, baroreceptors, brainstem rhythms, central pacemaker activity, cranial rhythmic impulse, cranial vault hold, electrocardiogram, heart rate variability, intermediate rhythm, osteopathic manipulative therapy, photoplethysmography, primary respiratory mechanism, respiration, skin perfusion

## Abstract

Cardiovascular rhythms representing functional states of the autonomic nervous system (ANS) are insufficiently reflected by the current physiological model based on low and high frequency bands (LF, HF, resp.). An intermediate (IM) frequency band generated by a brainstem pacemaker was included in systemic physiological ANS analyses of forehead skin perfusion (SP), ECG, and respiration. Data of 38 healthy participants at T0 and T1 (+1 week) before, during, and following osteopathic cranial vault hold (CVH) stimulation were analyzed including momentary frequencies of highest amplitude, amplitudes in low (0.05–0.12 Hz), IM (0.12–0.18 Hz), and high (0.18–0.4 Hz) frequency bands, and established heart rate variability (HRV) metrics. During CVH, LF interval durations increased, whereas IM/HF band durations decreased significantly. Amplitudes increased significantly in all frequency bands. A cluster analysis found one response pattern dominated by IM activity (47% of participants) with highly stable 0.08 Hz oscillation to CVH, and one dominated by LF activity (0.10 Hz) at T0, increasing to IM activity at T1. Showing frequency ratios at ≈3:1, respiration was not responsible for oscillations in PPG during CVH. HRV revealed no significant responses. Rhythmic patterns in SP and respiration matched previous findings on a reticular “0.15 Hz rhythm”. Involvement of baroreflex pathways is discussed as alternative explanation.

## 1 INTRODUCTION

The scientific study of physiological rhythms has been marked by a long history of controversies (Berntson et al., 1997; Camm et al., 1996; Koepchen, 1962). Opposing views interpreted cardiovascular rhythms either as “clanking of the cogs” or attributed them to vital functions indexing autonomic nervous system (ANS) activity (Laborde et al., 2017; Malpas, 2002; Park & Thayer, 2014; Pomeranz et al., 1985). Few fruits of this vast body of research have made it into clinical care of patients such as cardiac‐gated neuromodulation of baroreflex and pain sensitivity (Thieme et al., 2022). Yet there is no doubt today that cardiovascular rhythms represent different functional states of the ANS. Controversies in this field are spurred by the complexity of responses of the ANS when adapting to seemingly uncountable internal and external triggers, for adaptation is at the core of ANS functioning. Any physiological model of ANS activity therefore needs to account for these dynamic properties.

Heart rate variability (HRV) used as an adaptive physiological index of overall health is commonly divided into low frequency (LF; 0.04–0.15 Hz) and high frequency (HF; 0.15–0.4 Hz) components (Camm et al., 1996; Laborde et al., 2017). While the HF band is widely agreed as a surrogate of the cardiac vagal tone, the composition of the LF band is a matter of contention. Supposed to contain sympathetic and parasympathetic activity (Akselrod et al., 1981), a LF/HF ratio often computed to index autonomic balance (Laborde et al., 2017) is equally fraught with ambiguity and has therefore frequently been subject to criticism (Berntson et al., 1997; Goldstein et al., 2011; Moak et al., 2007; Reyes del Paso et al., 2013). In this respect, studies demonstrating nonstationary and nonlinear dynamics of HRV (Billman, 2013; Heathers, 2012; Malpas, 2002) clearly underpin the relevance to abandon the current concept for a more dynamic “second‐generation model” for ANS research.

The basics of such a model have been laid first with findings on a rhythm at approximately 0.15 Hz in unspecific reticular neurons (retR) in the common brainstem system of dogs (Lambertz et al., 2000; Lambertz & Langhorst, 1998; Langhorst et al., 1984). First evidence of this 0.15 Hz rhythm band in human (0.12–0.18 Hz, Ziege, 1992) earlobe and forehead skin perfusion (SP) (Perlitz, Lambertz, Cotuk, et al., 2004; Ziege, 1992) were extended to cardiac, respiratory, and blood pressure (BP) data in individuals experienced in the practice of autogenic training (AT; Perlitz, Cotuk, Lambertz, et al., 2004; Perlitz, Cotuk, Schiepek, et al., 2004). Furthermore, this rhythm band was observed to coordinate at integer number ratios, the so‐called n:m coordination. With the “0.15 Hz rhythm band” at its center, physiological signals may exhibit coupling within the IM range (1:1 ratio) or with upper (HF) and lower (LF) harmonics (1:2 or 2:1 ratios).

**FIGURE 1:**
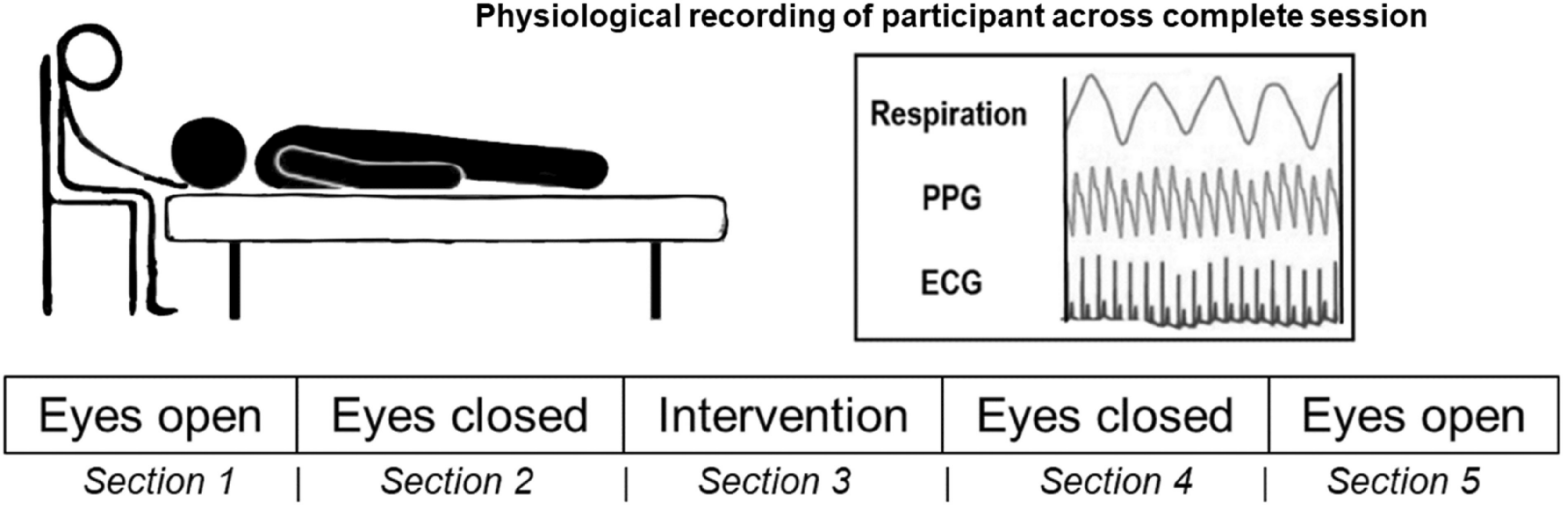
Experimental paradigm. Each session was comprised of five sections, lasting 300 s each. Section 1 and 5 (eyes open) as well as section 2 and 4 (eyes closed) were performed “hands‐off”. Section 3 was “hands‐on” during which the cranial vault hold was applied and participants' eyes were closed. During each session, respiration, forehead skin blood oscillations (PPG), and electrocardiogram (ECG) were recorded.

A recent trial using fMRI and peripheral physiological measures (respiration rate and HRV) found activity in the intermediate (IM) band (previously labeled “0.15 Hz rhythm band”) associated with activation in core interoceptive areas of the central autonomic network (Keller et al., 2020). This has expanded the previously held perspective of psychophysical relaxation as the most prominent psychological correlate of cutaneous IM band activity. It suggests IM band activity in skin microcirculation also be linked with central autonomic functioning. Labelling large spindle shaped oscillations, so‐called beat oscillations in photoplethysmographic recordings as “psychomotor” waves in the early days of this technology (Deutsch, 1952) may be an expression of this linkage. From the physics perspective, these large oscillations originate usually from interference of two adjacent rhythms. These oscillations have been also observed during AT in time series of respiration and inter‐beat‐intervals (see Figure 1a–c in Perlitz, Lambertz, Cotuk, et al., 2004).

Controversies and inconsistencies in the physiology of current ANS related frequency band concepts precipitated also in scientific studies in adjacent medical disciplines. A well‐known and highly controversial example for ongoing dispute is given with osteopathy in the cranial field (OCF). OCF, cranial osteopathic manipulative medicine, craniosacral osteopathy or originally the cranial‐sacral treatment (CST) developed by W.G. Sutherland some 90 years ago has at the center a palpable physiological phenomenon, the primary respiratory mechanism (PRM), termed also as “breath of life”, “tide” or, as of 1961, referred to as “cranial rhythmic impulse” (CRI) (Woods & Woods, 1961), which is supposed to represent a manifestation of the PRM. The CRI has been recorded successfully using various protocols with traditional palpation (for a review on this literature, see Sergueef et al., 2011) and despite some inconsistent results, several studies on immediate and long‐term effects of OCF/CST on ANS functioning have reported an increase of HF‐HRV parameters as modulation of parasympathetic activity (Arienti et al., 2020; Carnevali et al., 2020; Cerritelli et al., 2020; Henley et al., 2008; Rechberger et al., 2019; Ruffini et al., 2015).

Yet, despite its significance in CST the PRM/CRI is scarcely understood as to its origin. This may at least in part have been the source of the controversy reflected by the plethora of interchangeable professional terms (OCF, CST, etc.) or etiological conceptual terms (PRM, CRI, etc.) (McPartland & Mein, 1997; Nelson et al., 2006). The review by McPartland & Mein and their hypothesis of a linkage between ANS and CRI called for basic research in this field, and was followed by several studies using Laser‐Doppler flowmetry (LDA) to record blood flow velocity producing evidence on striking parallels of the CRI and Traube‐Hering‐Mayer (THM) waves (Nelson et al., 2001, 2006; Nelson et al., 2004). Depending on ANS activity in states considered a balance between sympathetic tone and parasympathetic tone, THM reflect changes in BP that vary on a beat‐to‐beat basis causing sinusoidal fluctuations of brain volume. This sympathetic‐parasympathetic balance has been shown to also induce a harmonic entrainment encompassing the multitude of oscillating tissues such as HRV, respiration rate, pulse transit time, and brain waves (McCraty et al., 1993; Tiller et al., 1996).

This notion of a sympathetic‐parasympathetic balance driven harmonic entrainment of the entire organism is apparently contradicted by recent functional magnetic resonance imaging (fMRI) studies reporting on a pacemaker based in the brainstem suggesting to split the LF‐band into a lower (LFa: 0.06–0.1 Hz) and an upper (LFb: 0.1–0.14 Hz) section (Pfurtscheller et al., 2021; Schwerdtfeger et al., 2020). Appearance of IM band activity in various peripheral signals as observed during AT may thus be triggered by reticular pacemaker activity and it may therefore index such harmonic entrainment, which has been known for long to be an outflow of central activity (Bethe, 1940).

Such large waves were triggered in LDA signals using standard OCF/CST techniques (see Figure 2 in Nelson et al., 2004). To date there is only limited literature available for understanding the effect of cranial vault hold (CVH) on the ANS as one of the most prominent “hands‐on” manual stimulation techniques used in OCF/CST (Cerritelli et al., 2020; Ferguson, 2003). Standard or CVH is an osteopathic technique commonly employed in the supine position to stimulate ANS activity by augmenting the PRM (Shi et al., 2011). CVH touches lateral cranial regions and cranial bones with the palm of both hands and the palmar surface of fingers (no thumb) at very low pressure on the scalp. Yet, the need to unravel the question of the origin of the PRM/CRI prevails as has been most recently stressed again (Bordoni & Escher, 2023).

It was therefore our goal to further the physiological evidence on a more dynamic physiological model based on inclusion of the IM 0.15 Hz rhythm band by external triggering of a relaxation response using OCF, similar to AT. The investigation across the entire range of LF, HF, and IM‐bands in cardiovascular and respiration signals appears a promising approach to the dynamics of ANS functioning.

In the current study, we probed systemic physiological ANS responses in photoplethysmography (PPG), electrocardiogram (ECG), and respiration activity in healthy individuals before, during, and following a specific OCF intervention, that is, CVH as a means established in OCF practice to trigger instantaneous profound psychophysical rest. Semi‐linear algorithms were used to compute LF, IM and HF band activity as combined time‐frequency parameters. We examined whether physiological reactions observed during CVH match those described for the CRI and whether these can be explained by IM band physiology to supply in‐depth understanding of the intricate physiology of LF‐, IM‐ and HF‐band interactions. We hypothesize that the physiological reactions in SP observed during CVH may be explained by a dynamic model of ANS functioning including changes in IM, LF and possibly HF band activity. We further wanted to explore similarities of IM band dynamics associated with CVH with those described during hypnoid states (Perlitz, Cotuk, Lambertz, et al., 2004).

## 2 MATERIALS AND METHODS

### 2.1 Experimental protocol and participants

Fifty‐three healthy adults participated in this study. Following exclusion of measurements due to extensive artifacts, technical or other problems, the final sample was comprised of 38 participants (23 female, 15 male, aged 40.7 ± 12.3 years). Whenever possible, examiners conducted two measurements with each participant (T0 and T1). In the final sample, 18 participants had completed two CVH measurements at an interval of 1 week (note: one T0 measurement was corrupted). Therefore, final analyses were performed with a total of 55 measurements and extended our previous analyses (preprint: Pelz et al., 2023). All participants were nonsmokers without osteopathic treatments during the preceding 3 months. Exclusion criteria were mental health symptomatology assessed with the ICD‐10 symptom rating (ISR) questionnaire (Tritt et al., 2010), a history of or acute neurological or cardiovascular disorders, current use of psychoactive medication as well as high‐performance sport, and smoking. Furthermore, to ensure spontaneous ANS activity, participants were instructed and formally agreed to abstain from caffeine for 4 h and from alcohol consumption for 48 h prior to testing. Recruitment was conducted through personal contact. Experimental sessions were held in private practice rooms of four osteopathic practitioners (examiner 1: *n* = 31 measurements; examiner 2: *n* = 14 measurements; examiner 3: *n* = 4 measurements; examiner 4: *n* = 6 measurements). The experiment was conducted according to the Code of Ethics of the World Medical Association (Declaration of Helsinki, 2008). All participants provided written, informed consent, and all protocols were approved by the Institutional Review Board of the state of Lower‐Saxony (EK vote from 04/03/2017).

### 2.2 Examiners

Four DGOM‐certified osteopaths participated in the study as examiners. At the time of the study, examiner A had been in private practice for 34 years and was also teaching OCF. Examiner A estimated that 60% of his patients would undergo some cranial treatment, whereas cranial treatment would be the major treatment regimen for approximately 50% of patients. Examiner B had been in private practice for 25 years and was also teaching OCF. Examiner B estimated that approximately 80% of his patients would receive at least some cranial treatment whereas this would be the major treatment for approximately 60% of patients. Examiner C and D have been active in a shared private practice for more than 20 years. Examiner C estimated that 70% of his patients received some cranial treatment and approximately 50% received cranial treatment as main treatment. Examiner D estimated that 80% of her patients received some cranial treatment and 60% received cranial treatment as main treatment.

### 2.3 Procedure

All participants underwent CVH as an osteopathic intervention commonly employed to stimulate ANS activity, preceded and followed by sections with open and closed eyes serving as within‐subject control conditions. Examiners and participants were blind to the data recording, and off‐line data analysis. As standard osteopathic hands‐on technique, CVH touches standardized cranial regions and cranial bones at very low pressure on the scalp. All examiners used Sutherland's CVH technique as described in Liem (2002). Examiners did not actively treat, but rather observed and augmented the CRI. Most participants reported a relaxed and calm perception of CVH. For the current communication the PPG, ECG as well as respiration signals of participants were used to study physiological rhythms before, during, and following OCF specific CVH intervention. During testing, the examiner was seated behind the head of the participant who remained in a supine position. Each experimental session was performed at comfortable room temperature and consisted of five consecutive sections (300 s each; following the first measurements of the study, 30 s were added between sections to account for motion artifacts produced during section transitions). These sections consisted of two different “hands‐off” resting states during which the participant was encouraged by an automated female voice to either keep the eyes open (EO) or eyes closed (EC) bracketing the “hands‐on” CVH phase during which eyes were closed as well (see Figure 1).

### 2.4 Physiological data acquisition and preprocessing

#### 2.4.1 Data acquisition

During the experimental procedure, physiological signals were recorded using PPG, ECG, and respiration activity. All recorded data was synchronized using timestamps. Sensors were time‐synchronized in the beginning of each measurement with a relative difference of less than 10 ms. the recorded data stream was transmitted to a local computer using Bluetooth and the complete measurement was uploaded to a private cloud for further offline processing. The PPG device used a standard Osram SFH7060 PG sensor able to emit red, infrared, and green light and simultaneously detect signal using a built‐in photodiode. The sensor was fixed at the participant's forehead glabella and care was taken to avoid cable contact with examiners during recordings. The data from this sensor was further processed with a microcontroller firmware which recorded the reflected red‐light signals at 660 nm at a sample rate of 125 Hz since we focused on ANS oscillations <0.4 Hz only. The red‐light signal was used to maintain comparability with previous studies (e.g., Perlitz et al., 2004). Ambient light was subtracted by IM measurements with all LEDs off. ECG data was recorded using a specialized analogue digital converter microchip (ADS1292) using a sampling frequency of 1000 Hz. Respiration was recorded with a sampling frequency of 500 Hz using a three‐axis accelerometer (ADXL354) attached to the chest (xiphoid process). The resulting respiratory signal was created as the magnitude of the x, y and z axis vector: $r=\sqrt{x^{2}+y^{2}+Z^{2}}$, where r represents the respiratory signal. For further processing, respiration data was downsampled to 125 Hz.

#### 2.4.2 Data denoising

Artifact removal was based on the application of stationary wavelet transform (SWT) (Borik et al., 2022), by which the raw signals were decomposed into the desired number of levels depending on the type of biosignal. For PPG and respiration data, a 12‐level decomposition was used, in which case the SWT can be considered as a band‐pass filter that also detrends the signal. In case of the PPG, only the approximation component was removed to preserve all signal details and, hence, this signal was only detrended with artifact suppression across the entire frequency band based on a moving standard deviation of 1 × σ. The sampling frequency was 125 Hz. A similar approach was used for respiration signals. However, only decompositions were selected so that the resulting signal contained only components from 0.03 Hz to 0.5 Hz. For ECG data, the sampling was left at the original 1000 Hz to preserve the morphological features of the ECG signal, especially fine located R‐wave position. For the SWT application, only an 8‐level decomposition was used, with suppression of the high‐frequency components and enhancement of the frequency components related to the QRS complex energy, ranging from approximately 2 Hz–32 Hz.

#### 2.4.3 Computation of physiological measures

Before signal analysis, physiological time series (PPG, ECG and respiration) were clipped to remove redundant segments. Furthermore, PPG and respiration data was downsampled to 5 Hz to improve performance of further data processing steps.

#### 2.4.4 Momentary frequency of highest amplitude (MFHA) for PPG and respiration

Offline processing of denoised PPG and respiration data was performed using Numpy (Harris et al., 2020), SciPy (Virtanen et al., 2020), and Matplotlib (Hunter, 2007). To further clear the time‐frequency plot from artifacts the signal was detrended. The preprocessed signal was then converted to a time‐frequency distribution using the continuous wavelet transformation and a Morlet wavelet (with parameter *σ* = 5) computed in relative normalization. The frequency ranged from 0.05 to 0.4 Hz and was sampled in 200 steps. Morlet wavelet time frequency distributions were computed in relative normalization, which displayed only frequencies of highest amplitude of the respective window. 3D‐TFD maps were then reduced to 2D‐time series of momentary frequencies of highest amplitude (MFHA), namely the frequency with the highest amplitude for each time step. This method is similar to the time‐frequency ridge function in MATLAB which extracts the instantaneous frequency of a signal using the Fourier transform. Frequency band interval durations (total of 300 s for each section) were computed from MFHA values and were comprised of a single dominating frequency for each time bin. These time bins were used to compute the interval durations in different frequency bands for each section. The MFHA was analyzed for distribution of frequency band activity in previously published defined frequency bands (LF: 0.05–0.12 Hz; IM: 0.12–0.18 Hz; HF: 0.18–0.4 Hz; Perlitz, Lambertz, Cotuk, et al., 2004).

#### 2.4.5 PPG signal amplitudes

Furthermore, a set of the algorithms implemented in Matlab R2022a (Mathworks Inc., Natick, MA) was used to extract signal amplitudes for each frequency band (LF: 0.05–0.12 Hz; IM: 0.12–0.18 Hz; HF: 0.18–0.4 Hz). Firstly, they were denoised from mostly motion artifacts using SWT and filtered using bandpass filters according to the defined frequency bands. Remaining peaks indicative of movement artifacts were suppressed using a soft thresholding function with a moving median (500 samples = 100 s) and 3 × σ. Finally, amplitudes were extracted for each frequency band from scalograms computed by continuous wavelet transformation. Extracted amplitudes were normalized by min‐max scaling for each subject. The min‐max normalization of the entire record (across all five phases of the experiment) but for each subject separately ensured comparability of ANS responses between subjects. In this way, we were able to track changes in PPG amplitude across experimental stages while minimizing measurement error caused by the different optical properties of each subject's tissue. That is, the amplitude of perfusion in a given frequency domain was detected independently and we only tracked its relative changes across the entire experiment. At the same time we could compare the entire cohort of subjects examined in this way.

#### 2.4.6 HRV metrics

HRV metrics were computed based on ECG data. All datasets were individually imported in Kubios HRV Scientific 4.0.2. Automatic noise detection (low) as well as automatic beat correction and visual inspection were applied before further analyses. It was ensured that the automatic beat correction for each experimental section did not exceed 5%. On average 0.28% of beats underwent automatic beat correction. Percentage of corrections was not significantly different between sections (*F*(4, 249) = 1.0, *p* = 0.39). HRV data were then exported for statistical analysis. Metrics included standard measures, namely the root mean square of successive differences (RMSSD), the power in LF, IM, and HF frequency bands, and mean heart rate (Shaffer & Ginsberg, 2017). The natural logarithm of power in LF, IM and HF frequency band was used as power values display a large skew.

#### 2.4.7 Statistical analyses

Linear mixed models were run for all physiological metrics (PPG mean MFHA, PPG MFHA frequency band durations, PPG amplitudes, HRV metrics, respiratory MFHA) as dependent variable as well as “section” (1–5), “examiner” (1–4) and “timepoint” (1 vs. 2) as fixed effects. Participant was added as random effect, including the random intercept. The model specification was as follows: Dependent variable ~ section + examiner + timepoint + (1|Participants). Significance was calculated based on Satterthwaite's method, estimating degrees of freedom and generating *p*‐values. In case of significant main effects, post hoc tests (Bonferroni correction) probed differences between individual sections, examiners or timepoints. Statistical analyses were performed in JASP (JASP Team (2023). JASP (Version 0.17.2) [Computer software]). A *p*‐value of <0.05 was considered statistically significant.

As exploratory analysis, LF, IM and HF duration changes from section 2 to CVH were computed [CVH—section 2] and were then submitted to a *K*‐means cluster analysis (Hartigan, 1975) to explore whether participants showed different types of physiological responses during CVH. This analysis was done using PSPP version 1.6.2 open‐source statistical software. Furthermore, mean MFHA frequencies for PPG and respiration per section were plotted for all participants as well as separately for the two groups, based on *K*‐means clustering analysis results.

**FIGURE 2:**
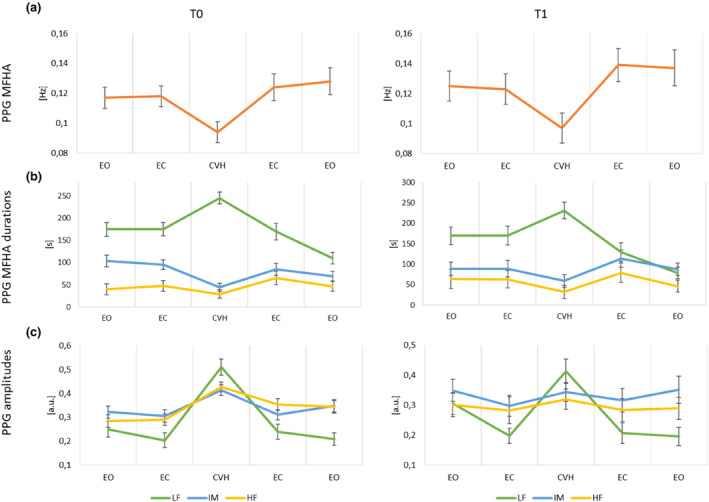
Group averages for T0 and T1 of (a) PPG mean frequencies of highest amplitude (MFHA) in [Hz] (see also Figure 3a), (b) PPG MFHA frequency band interval durations in [s] for LF‐, IM‐ and HF‐bands (green, blue, and yellow, resp.), and (c) PPG amplitudes in [a.u.] for LF‐, IM‐ and HF‐bands (green, blue, and yellow, resp.). Plots show significant increases of LF‐ and significant decreases of IM‐ and HF‐band interval durations during cranial vault hold (CVH). There is a significant main effect of timepoint with shorter LF interval durations at T1 compared to T0 and longer HF interval durations at T1. LF amplitudes were significantly higher during CVH compared to all other sections. IM amplitudes were significantly higher during CVH compared to eyes closed sections, and significantly higher for CVH compared to section 1 and 2 for HF amplitudes. HF amplitudes were significantly lower during T1 compared to T0. Sections: 1. eyes open (EO); 2. eyes closed (EC); 3. EC + CVH; 4. EC’; 5. EO’. HF, high frequency; IM, intermediate; LF, low frequency.

## 3 RESULTS

### 3.1 PPG data

#### 3.1.1 MFHA and frequency band durations

The dominating frequency in skin microcirculation, that is, that of highest amplitude was probed for each experimental section (Table 1; Figure 2a). A linear mixed effects model for the MFHA showed a significant effect of “section” (*F*(4, 230.3) = 12.8, *p* < 0.001) as well as “timepoint” (*F*(1, 248.2) = 19.1, *p* < 0.001), but not for “examiner” (*F*(3, 33.8) = 0.4, *p* = 0.74). Bonferroni‐corrected post hoc tests revealed a significant difference between CVH and all other sections (*p* < 0.001), whereas the changes from section 1 to 5 (*p* = 0.06) as well as from section 2, 4 (*p* = 0.09) were not significant.

**TABLE 1:** Descriptives and main effects of PPG mean of momentary frequency of highest amplitude (MFHA) linear mixed effects models.

Fixed effect		Descriptives	ANOVA fixed effects		
		MFHA (M ± SD)	*F*	df	*p*
Section	EO	0.120 (±0.04)	12.8	4, 230.3	**< 0.001**
	EC	0.119 (±0.04)			
	CVH	0.096 (±0.04)			
	EC	0.129 (±0.05)			
	EO	0.132 (±0.05)			
Examiner	1	0.118 (±0.05)	0.4	3, 33.8	0.74
	2	0.116 (±0.04)			
	3	0.115 (±0.02)			
	4	0.135 (±0.06)			
Timepoint	T0	0.116 (±0.05)	19.1	1, 248.2	**< 0.001**
	T1	0.125 (±0.05)			

Frequency band averaged interval durations computed from MFHA values displayed a single dominating frequency for each time bin. Therefore, the sum of durations for LF, IM, and HF amounted to 300 s per section (see Figure 2b; Table 2). Linear mixed effects models revealed a significant effect of “section” for LF (*F*(4, 230.9) = 31.6, *p* < 0.001), IM (*F*(4, 232.7) = 8.1, *p* < 0.001), and HF (*F*(4, 230.7) = 4.5, *p* < 0.01) interval durations. The effect of “examiner” was not significant for any model (>0.15). However, there was a significant effect of “timepoint” for the LF band (*F*(1, 257.3) = 10.3, *p* = 0.001) as well as the HF band (*F*(1, 249.8) = 10.2, *p* < 0.01), while there was no timepoint effect for the IM band (*F*(1, 261.6) = 1.6, *p* = 0.2). Bonferroni‐corrected post hoc tests showed that mean frequency interval durations of LF activity were significantly longer in section 3 (CVH) compared to all other control sections (all *p* < 0.001) as well as a significant change from EC1 to EC2 section (*p* < 0.001). Furthermore, IM interval durations were significantly shorter during CVH than all other control sections (IM: CVH vs section 5: *p* = 0.03, all others: *p* < 0.001). For the HF band, this effect was only found for the contrast between CVH and section 4 (*p* < 0.001). The effect of “timepoint” revealed significantly shorter LF interval durations at T1 (155.7 ± 101.4) compared to T0 (174.6 ± 100.1), whereas HF interval durations were significantly longer during T1 (56.1 ± 83.8) compared to T0 (44.8 ± 70.4).

**TABLE 2:** Descriptives and main effects of interval duration of PPG momentary frequency of highest amplitude (MFHA) frequency band linear mixed effects models.

Fixed effect		Descriptives			ANOVA fixed effects			
		LF (M ± SD)	IM (M ± SD)	HF (M ± SD)	Band	*F*	df	*p*
Section	EO	172.9 (±92.1)	99.0 (±74.4)	48.0 (±85.1)	LF IM HF	31.6 8.1 4.5	4, 230.9 4, 232.7 4, 232.3	**<0.001** **<0.001** **<0.001**
	EC	173.6 (±92.9)	92.9 (±74.4)	53.1 (±79.3)				
	CVH	240.3 (±82.1)	49.2 (±58.4)	30.2 (±61.1)				
	EC	155.5 (±107.7)	94.4 (±84.6)	69.7 (±93.8)				
	EO	99.0 (±75.4)	75.3 (±65.5)	46.6 (±66.2)				
Examiner	1	168.7 (±97.9)	82.2 (±70.4)	48.9 (±80.3)	LF IM HF	0.3 1.9 0.7	3, 33.9 3, 35.3 3, 34.1	0.82 0.18 0.54
	2	167.9 (±109.0)	84.5 (±85.4)	47.1 (±65.9)				
	3	161.3 (±83.4)	130.8 (**±**66.6)	7.8 (±10.6)				
	4	171.3 (±110.4)	42.9 (**±**41.5)	86.1 (±104.8)				
Timepoint	T0	174.9 (±100.1)	79.1(±71.9)	45.7 (±73.2)	LF IM HF	10.3 1.6 10.2	1, 257.3 1, 261.6 1, 249.8	**0.001** 0.21 **<0.01**
	T1	155.7 (±101.4)	87.5 (±77.5)	56.8 (±87.6)				

### 3.2 Psychophysiological reactions during CVH—MFHA clustering analysis

Mean MFHA of all participants showed a decrease from 0.12–0.13 Hz at baseline to 0.095 Hz during CVH. An explorative *K*‐means clustering analysis based on LF, IM and HF duration change from the mean of section 2 (EC1) to CVH, showed two distinct response patterns to CVH. Part of the sample exhibited a pattern of CVH IM activity before and after CVH (“IM‐responders”) as well as an immediate significant decrease from IM activity to activity in the LF range (*N* = 26; mean duration change EC to CVH: LF = 136.8, IM = − 80.1, HF = −56.7). Another group showed a pattern (“LF‐responders”) which started and remained in LF activity at T0 and exhibited no response to CVH besides a slight increase of PPG frequencies over time (*N* = 29; mean change: LF = 3.8, IM = −11.1, HF = 7.3). At T1, PPG frequencies increased to IM activity following CVH.

**FIGURE 3:**
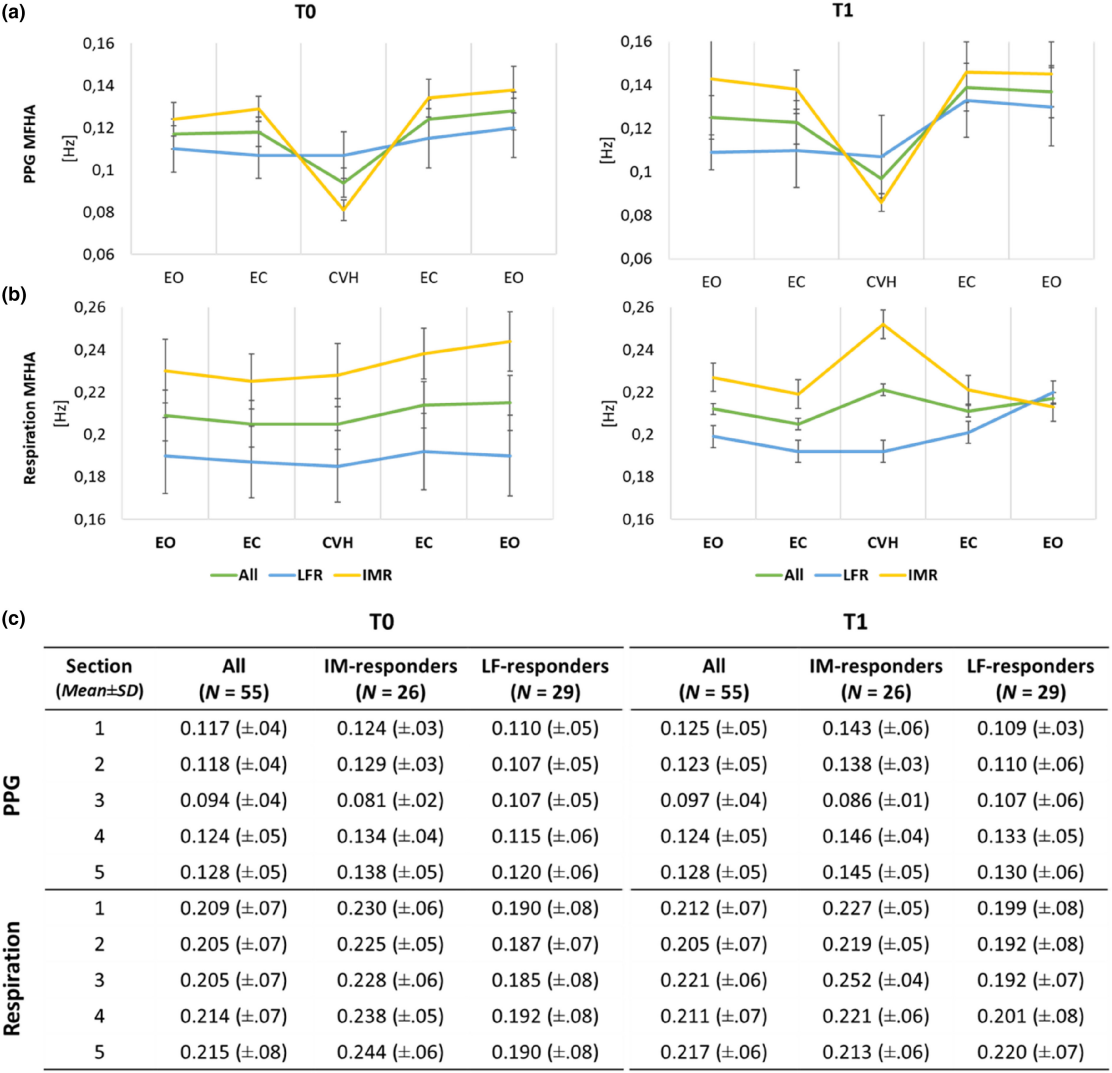
Averaged momentary frequency of highest amplitude (MFHA) derived from Morlet Wavelet time frequency distribution in absolute normalization of recordings of (a) forehead skin PPG and (b) respiration for T0 and T1. (c) shows the descriptives for (top) PPG and (bottom) respiration data. Line graph (top) and descriptives (bottom) for the whole sample (all) and clustering‐based LF‐responders (LFR) and IM‐responders (IMR). Error bars in plots show standard errors of the mean.

Averaged MFHA time courses for all participants as well as for separate groups showed that “LF‐responders” remained in LF mode, while the decrease of “IM‐responders” to 0.08 Hz during CVH revealed a stable low oscillation mode. See Figure 3 for group time‐courses and descriptive statistics.

### 3.3 PPG signal amplitudes

ANS activity also affects amplitude dynamics on the cardiac level and on the level of the vasculature (for respiratory amplitude responses see below). We therefore compared changes in SP amplitude along the boundaries of LF, IM, and HF bands computing linear mixed effects model with section, examiner and timepoint as fixed effects as well as the random intercept of participants (Figure 2b; Table 3). There was an overall significant difference of amplitudes between sections for the LF band (*F*(4,229.8) = 30.3, *p* < 0.001), the IM band (*F*(4,232.8) = 3.5, *p* < 0.01), and the HF band (*F*(4,232.7) = 5.1, *p* < 0.001). Post hoc Bonferroni corrected pairwise comparisons showed significantly higher amplitudes during CVH compared to all other sections in the LF band (all *p* < 0.001), between EC sections and CVH for IM‐band amplitudes (*p* < 0.05), and between sections 1/2 and CVH for HF‐band amplitudes (*p* < 0.001. Differences between EO and EC sections were not significant for any frequency band (all *p* > 0.2). There was a significant main effect of examiner for LF and IM amplitudes (LF: *F*(3,29.7) = 6.1, *p* < 0.01; IM: *F*(3,33.4) = 3.2, *p* < 0.05). Post hoc tests between all examiners showed a significantly different LF amplitude for examiners 1 and 2 (*p* < 0.05), examiners 2 and 3 (*p* < 0.05), as well as between examiners 2 and 4 (*p* = 0.001). However, for IM amplitudes there was only a marginally significant difference between examiner 2 and 3 (*p* = 0.056). The dynamic pattern of amplitude increase during CVH was similar for all examiners.

**TABLE 3:** Descriptives and main effects of PPG amplitude linear mixed effects models.

Fixed effect		Descriptives			ANOVA fixed effects			
		LF (M ± SD)	IM (M ± SD)	HF (M ± SD)	Band	*F*	df	*p*
Section	EO	0.269 (±0.18)	0.331 (±0.15)	0.290 (±0.16)	LF IM HF	30.3 3.5 5.1	4, 229.8 4, 232.8 4, 232.7	**<0.001** **<0.01** **<0.001**
	EC	0.202 (±0.17)	0.303 (±0.15)	0.288 (±0.17)				
	CVH	0.476 (±0.20)	0.390 (±0.14)	0.390 (±0.14)				
	EC	0.228 (±0.18)	0.313 (±0.14)	0.330 (±0.15)				
	EO	0.205 (±0.15)	0.349 (±0.17)	0.327 (±0.16)				
Examiner	1	0.277 (±0.20)	0.346 (±0.14)	0.325 (±0.16)	LF IM HF	6.1 3.2 0.9	3, 29.7 3, 33.4 3, 34.3	**<0.01** **<0.05** 0.44
	2	0.207 (±0.22)	0.289 (±0.19)	0.295 (±0.17)				
	3	0.342 (±0.17)	0.402 (±0.16)	0.323 (±0.12)				
	4	0.390 (±0.13)	0.361 (±0.11)	0.395 (±0.16)				
Timepoint	T0	0.282 (±0.22)	0.340 (±0.15)	0.340 (±0.15)	LF IM HF	0.6 0.7 4.6	1, 265.0 1, 265.9 1, 265.8	0.44 0.42 **<0.05**
	T1	0.265 (±0.17)	0.332 (±0.16)	0.296 (±0.17)				

Timepoint of measurement had only a significant effect for the HF range with higher amplitudes during T0 (*F*(1, 265.8) = 4.6, *p* < 0.05).

### 3.4 ECG data

#### 3.4.1 CVH effects on HRV measures

The effect of EO, EC and CVH was investigated in a range of standard HRV measures based on ECG data. HRV measures included RMSSD, the natural logarithm of power in different frequency bands (LF, IM and HF), as well as mean HR. Linear mixed effects models were run for the obtained HRV metrics using “section”, “examiner” and “timepoint” as fixed effects as well as the intercept of participant as random effect (see Table 4).

**TABLE 4:** Descriptive statistics (mean ± SD) of HRV metrics for fixed effects section, examiners, and timepoint.

	Section					Examiner				Timepoint	
Measure	EO	EC	CVH	EC	EO	1	2	3	4	T0	T1
RMSSD	39 ± 22	36 ± 20	39 ± 23	40 ± 23	39 ± 22	38 ± 24	45 ± 18	30 ± 9	14 ± 6	37 ± 22	42 ± 22
LnLFpower	5.96 ± 1.33	5.66 ± 1.30	5.77 ± 1.25	5.90 ± 1.22	5.93 ± 1.28	5.71 ± 1.28	6.15 ± 1.16	6.47 ± 1.07	4.36 ± 0.59	5.78 ± 1.22	5.98 ± 1.36
LnIMpower	4.94 ± 1.56	4.99 ± 1.61	4.87 ± 1.42	5.08 ± 1.51	5.07 ± 1.65	4.73 ± 1.61	5.73 ± 1.15	5.37 ± 0.84	2.76 ± 0.39	4.87 ± 1.51	5.24 ± 1.58
LnHFpower	5.55 ± 1.41	5.49 ± 1.32	5.54 ± 1.38	5.63 ± 1.41	5.53 ± 1.32	5.53 ± 1.48	6.08 ± 0.89	4.71 ± 0.87	3.87 ± 0.65	5.46 ± 1.39	5.72 ± 1.28
MeanHR	63.8 ± 9.7	63.5 ± 9.7	61.5 ± 8.9	61.8 ± 8.9	62.6 ± 8.8	63.3 ± 9.8	61.7 ± 7.9	60.1 ± 10.1	65.5 ± 4.3	64.2 ± 9.1	59.5 ± 8.6

The analysis of RMSSD did not reveal any significant effect (section: *F*(4,202.0) = 1.5, *p* = 0.21; examiner: *F*(3, 34.1) = 1.4, *p* = 0.27; timepoint: *F*(1, 212.5) = 2.4, *p* = 0.12). Therefore, no post hoc tests were performed. For the natural log of LF power, there was also no significant change related to section (*F*(4,202.2) = 2.3, *p* = 0.06), examiner (*F*(3, 34.4) = 2.0, *p* = 0.13) or timepoint (*F*(1, 217.6) = 2.1, *p* = 0.15). The natural log of IM power, however, showed a significant effect of examiner (*F*(3, 34.3) = 3.3, *p* < 0.05) while fixed effects section (*F*(4,202.2) = 0.9, *p* = 0.48) and timepoint (*F*(1, 217.1) = 0.3, *p* = 0.60) remained nonsignificant. Bonferroni‐corrected post hoc tests for examiners showed only a significant difference between examiner 2 and 4 (*p* < 0.05) with lower IM power in participant measured by examiner 4. The HF power did not reveal any significant effect (section: *F*(4,202.0) = 0.6, *p* = 0.65; examiner: *F*(3, 34.1) = 2.5, *p* = 0.08; timepoint: *F*(1, 210.9) = 2.2, *p* = 0.14). Interestingly, mean heart rate showed a significant effect of section (*F*(4,201.8) = 5.5, *p* < 0.001) as well as for timepoint (*F*(1, 207.6) = 4.0, *p* = 0.05), but not for examiner (*F*(3, 33.8) = 0.1, *p* = 0.96). Bonferroni‐corrected post hoc tests for section showed a significant decrease of mean HR from section 1 to CVH (*p* < 0.001), section 2 to CVH (*p* < 0.01), as well as section 2, 4 (*p* < 0.05). Further, mean HR was lower for T1 compared to the first measurement T0.

### 3.5 Respiration data

#### 3.5.1 Respiratory frequencies derived from mean frequency of highest amplitude

To exclude possible effects of respiratory activity on PPG amplitude and frequency interval duration, the MFHA was computed for respiration for all participants as well as based on our exploratory clustering analysis (Figure 3b). A linear mixed effects model showed that experimental section (*F*(4,231.0) = 0.8, *p* = 0.53) as well as timepoint of measurement (*F*(1,243.7) = 0.4, *p* = 0.53) had no significant effect on respiratory frequency for the entire sample. However, there was a significant trend effect of examiner (*F*[3,34.63] = 3.6, *p* = 0.02) with lower respiratory frequencies in participants treated by examiner 3 compared to examiner 1 and 2 (*p* < 0.05; examiner 1: 0.21 ± 0.07; examiner 2: 0.22 ± 0.07; examiner 3: 0.12 ± 0.03; examiner 4: 0.25 ± 0.04).

## 4 DISCUSSION

In the present study we extended the currently employed physiological low frequency (LF)‐ and high frequency (HF) concept by an intermediate (IM) band. Wedged, as it were, between the two former, the IM band has facilitated the investigation of peripheral time series such as obtained when monitoring effects of a standard cutaneous cranial osteopathic treatment known to evoke systemic ANS responses (Nelson et al., 2001, 2006; Nelson et al., 2004; Rechberger et al., 2019; Shi et al., 2011). Since the systemic study of organismic effects has been suggested to include recordings of at least two weakly coupled systems (von Bonin et al., 2014), we investigated physiological ANS responses in photoplethysmographic (PPG) recordings of forehead skin microcirculation, ECG, and respiration in 38 healthy non‐symptomatic adults continuously before, during and following CVH applied by four experienced osteopathic practitioners under identical study conditions. The recorded physiological signals were examined using time‐frequency analyses with high temporal resolution (Pelz et al., 2023). This allowed relating time‐synchronized outcomes to the following frequency scheme of systemic ANS variability in LF (0.05–0.12 Hz, 3–7.2 cpm), IM (0.12–0.18 Hz, 7.2–10.8 cpm), and HF (0.18–0.4 Hz, 10.8–24 cpm) bands (Keller et al., 2020; Perlitz, Cotuk, Lambertz, et al., 2004) as well as to standard HRV measures (Shaffer & Ginsberg, 2017).

### 4.1 Momentary frequency of highest amplitude (MFHA) in PPG

We computed the MFHA of PPG signals for each experimental section assuming the dominating frequency be the one most likely palpated by our osteopathic practitioners. Frequencies initially palpated for the CRI/PRM were first shown by Woods and Woods (1961) at 10–14 cpm (0.16–0.23 Hz). These subjectively recorded rates were later corrected by objective instrumentally recorded CRI/PRM data using LDA during standard CVH. Rates thus obtained were between 0.1–0.15 Hz, which is not only well in keeping with traditional THM waves (Nelson et al., 2006) but also with currently reported pacemaker activity in the human brainstem (Pfurtscheller et al., 2021; Schwerdtfeger et al., 2020). Transcranial bioimpedance recordings of intracranial fluid oscillations demonstrated rates exerted by cranial stimulation between 0.12–0.15 Hz (Moskalenko & Kravchenko, 2004). In our previous publication, we were able to demonstrate that successful palpation of the CRI/PRM was in phase with respective frequencies in MFHA (Pelz et al., 2023). During CVH, the averaged MFHA in PPG data decreased significantly from 0.119 Hz (upper LF band) to 0.096 Hz, and following CVH the averaged MFHA increased significantly to 0.129 Hz (IM band). At T0, the averaged MFHA of all recordings were at 0.116 Hz, and at T1 averaged MFHA of all recordings were at 0.125 Hz. This increase was significant (*p* < 0.001). This does reflect a distinct shift of ANS activity from LF band activity to IM band activity in several participants. However, it is important to note that these values have been averaged for each section and across participants. Since biological systems are affected by numerous factors, they cannot be mathematically exact. Borders of defined frequency schemes may therefore pose problems and should be considered with care. In order to obtain more comprehensive insight into the physiological mechanism, other parameters ought to be examined such as their systemic interaction (e.g., cardiorespiratory coordination). Employing linear mixed effects models, we found the duration of the MFHA increased significantly in response to CVH in LF, whereas it decreased in IM and in HF bands. From T0 to T1 there was a significant decrease of LF durations and a significant increase of HF durations.

### 4.2 Exploratory *K*‐means clustering analysis of PPG MFHA data

When exploring the physiological data, we observed that some participants responded with a distinct shift from IM band activity to an increase in the activity and duration of LF MFHA, while other participants remained mostly within an LF mode shown initially. A respective exploratory *K*‐means clustering analysis based on the MFHA duration change from section 2 to CVH thus revealed two distinct clusters. In the first cluster, participants exhibited predominantly IM frequency responses (IMR; *N* = 26) in EO and EC sections (i.e., sections 1, 2, 4, and 5). MFHA plots show that they responded to CVH with a steep and immediate decline from IM band at 0.129 Hz–0.081 Hz within the lower LF band, which increased significantly in duration (+136.8 s). This increase in duration was apparently balanced by a concomitant decrease of IM duration (−80.1 s) and HF duration (−56.7 s). This suggests a shift of ANS activity between these three frequency bands. In the second cluster, participants showed at T0 mostly LF frequencies (LFR; *N* = 29). In LFR there was no change during CVH at T0 (section 2 and 3 each at 0.107 Hz). Only in section 5 did frequencies reach lowest IM level at 0.12 Hz. At T1, following CVH, LFR showed a distinct increase of ANS activity from LF frequencies to IM frequencies. In the course of T1, frequencies rose from 0.109 to 0.13 Hz (section 1 and 5). Thus, there were increases in frequencies in both clusters reaching all IM levels. Applying the dichotomous LF‐HF concept to these data would have been concealed (LF: 0.04–0.15 Hz) such developments. Use of a triple‐band frequency concept allowed the detection and differentiation of ANS responses to the IM band physiology. Splitting the LF band into a section below and above the 0.1 Hz component, a lower LFa (0.06–0.1 Hz) and an upper LFb (0.1–0‐14 Hz) band, has been suggested recently to account for MRI scanning related anxiety processing. This finding is in line with earlier reports on two harmonically unrelated timely stable spectral components in HRV and systolic arterial BP, one band at 0.076 Hz (±0.012), the other at 0.117 Hz (±0.016) attributed both to baroreflex control of BP (Kuusela et al., 2003). While this notion had been supported by research on brain‐heart interaction (Pfurtscheller et al., 2021; Schwerdtfeger et al., 2020) these authors readily conceded that the upper LF band (0.1–0.14 Hz) lacks scientific study. ANS responses in our data on SP prior, during, and following CVH may be explained by various physiological pathways, the most probable ones being IM band physiology and central autonomic network inputs including efferences from various baroreflex activity.

### 4.3 IM band approach

Following the notion of integer number coordination associated with IM band dynamics, this significant increase of durations of frequencies and the decline to the lower LF band may be interpreted as subharmonic oscillations of the previously prevailing IM band activity (Perlitz, Cotuk, Lambertz, et al., 2004). This interpretation is supported by a conspicuous loss of variance or, conversely, by a gain in stability during CVH. This finding might indicate synchronization with another source of rhythmic activity such as respiration. This notion is supported by our findings for frequencies in respiration and PPG approaching 1:3 ratios. Given the shift from LF band to IM band between T0 and T1 with a distinct decline of frequencies during CVH may indicate coupling or lower harmonic activity of a pacemaker, such as the one described for unspecific neurons in the reticular formation (Lambertz et al., 2000; Lambertz & Langhorst, 1998). Recent findings in fMRI studies confirmed pacemaker activity in the left human brainstem with activities at 0.1 and 0.15 Hz attributing the 0.15 Hz rhythm to R‐to‐R intervals and respiration. Both pacemaker activities were observed separately in various individuals, as well as within a single subject (Schwerdtfeger et al., 2020). We concede, though, that we have previously assessed coupling in physiological data mostly employing casuistic data. Averaging data as applied in the present study may conceal, suspend, or falsely insinuate physiological mechanisms. In our data, averaging may have concealed n:m integer number coordination. Here, IMR results showed at T0 in section 2 averaged frequencies of 0.129 Hz ± 0.03, at section 3 (CVH) averaged frequencies of 0.081 Hz ±0.02. The lower value is not exactly a subharmonic, but given the standard deviations, n:m coupling could indeed have been present. Moreover, one has to bear in mind that synchronization in biological systems will not always adhere to strict mathematical or physical rules of precision but instead will always bear some degree of vital variability due to ongoing adaptation dynamics (Lambertz & Langhorst, 1998). Loss of such vital variability has been observed as a hallmark in epileptic seizures (Wallois et al., 2008). Prolonged phase‐locking has also been shown to index pathological conditions such as sleep apnea (Riedl et al., 2014). Therefore, the respiratory frequency may well play an important role during CVH. However, PPG waves in CVH may be related to n:m synchronization under central influence between both physiological signals rather than being caused by declined ANS activity and concomitant slowing of respiration. For a more elaborate and sound conclusion further analyses including coordination and/or synchronization ought to be conducted.

The decrease of frequencies to the LF range during CVH may also indicate increased sympathetic activity since microneurography recordings in non‐symptomatic participants have confirmed that frequencies up to 0.1 Hz can be sympathetically modulated (Stauss et al., 1998, 1999; Zegarra‐Parodi et al., 2014). This is supported also by findings that the lower LF band being sensitive to vascular mechanisms of sympathetic origin (Friedman, 2007; Schwerdtfeger et al., 2020). This might also explain the apparent reduction of variance during CVH at a mean frequency of 0.081 Hz. As becomes evident when inspecting single original recordings, this loss in variance is not the result of averaging but is a prominent feature in each single record.

As mentioned above, this finding may yet be linked to baroreceptor circuitry. These baroreceptors are linked in multiple ways with higher neural pathways, such as the ascending reticular activating system (ARAS), but they also play a vital role in central peripheral organs. Thus, carotid and cardiopulmonary baroreceptor reflexes are known to modulate autonomic output thereby maintaining resting BP, buffering excessive fluctuations in arterial pressure (carotid sinus baroreceptors), and controlling intravascular volume via cardiopulmonary baroreceptors (Suarez‐Roca et al., 2019). Stimulation of baroreceptors following increases in BP reach the dorsal medial nucleus of the solitary tract (dmNTS) reflex arcs, which, in turn, modulates BP, heart rate (HR), vascular dilation, autonomic balance, sleep, and pain perception. These physiological pathways have been successfully applied to the treatment of chronic pain in fibromyalgia (Thieme et al., 2022).

The frequencies (0.129 and 0.081 Hz) found particularly in our LMR cluster at T0 and those reported in harmonically unrelated timely stable spectral components in HRV and systolic arterial BP (Kuusela et al., 2003) apparently match. They may, therefore, be considered an outflow of baroreceptor activation since the dmNTS and the reticular formation are closely connected via afferent control (Lambertz et al., 2000). As the CVH stimulation ceases, activity seemed to have shifted towards the reticular formation activating also interoceptive networks, as becomes evident with increases in all frequencies following CVH in T0 and T1.

Systemic responses indicating central nervous system activity have also been documented for activity in the skin at 0.15 Hz (Keller et al., 2020; Perlitz, Lambertz, Cotuk, et al., 2004; Ziege et al., 1997). In particular, this included synchronization at integer number ratios with respiration, which is suggested also by our current findings. Such physiological response modalities are essential constituents of the power of ANS mediated adaptation, which aims at economizing the modes of operation of physiological systems (Bethe, 1940; McCraty et al., 2009; Mirollo & Strogatz, 1990; von Holst, 1939).

### 4.4 Respiratory MFHA—Involvement of respiration during CVH

One may hypothesize that the distinct decline in PPG oscillations during CVH may be related to a respective decline in respiratory frequencies since respiration is known to trigger related oscillations in the vasculature (Ziege et al., 1997). The general pattern observed in our respiratory MFHA data, however, clearly suggests that respiratory activity is not the source of CVH related LF or IM oscillations in forehead SP. For LFR, the respiratory frequency remained stable across experimental conditions at T0 and T1. Furthermore, at T0 the IMR also showed stable respiratory frequencies whereas there was an increase of frequencies during CVH and a decline towards sections 4 and 5 at T1. For IMR, the standard deviations during CVH suggest possible coupling between PPG and respiration at T0 (respiration: 0.228 Hz ± 0.06, PPG: 0.081 Hz ± 0.02;) and at T1 (respiration: 0.252 Hz, PPG: 0.086 Hz ± 0.01), which differed both approximately by factor 3. This is in support of the aforementioned integer number synchronization (n:m coordination) described for PPG, HRV, respiration, and BP as a phenomenon triggered by hypnoid relaxation using autogenic training (Perlitz, Cotuk, Lambertz, et al., 2004).

### 4.5 PPG amplitude responses

To deepen our understanding of the physiological adaptation processes during CVH, PPG amplitude changes across experimental sections were included in this investigation. There were significant increases in amplitudes in the LF, IM, and in HF bands during CVH compared to all other sections. The prominent frequency decrease in MFHA to 0.08 Hz activity was associated with distinct increases in amplitudes appearing as spindle shaped oscillations in the raw PPG signals. Early studies had already described spindle‐shaped oscillations in SP (Deutsch, 1952), which were then referred to as psychomotor waves. These phenomena are also known as beat oscillations which may originate from interference of closely coupled rhythmic sources (Perlitz, 2021). Such oscillators or pacemakers have been identified in the brain stem and they are hallmarks of IM band activity (Rassler et al., 2018). Here, we also observed a significant effect of examiner with differences in LF and IM band amplitudes. The beat oscillations might have resulted from adjacent brainstem‐based pacemakers in the dmNTS and the reticular formation. Interestingly, all examiners displayed a similar pattern of amplitude increase during CVH, however, starting at different baseline levels. Such reciprocal response patterns for frequencies and amplitudes (frequency ↑, amplitude ↓, and vice versa) are known for the force‐frequency product in cardiac physiology as negative staircase. Notably, we did not observe any significant effects in amplitude responses due to EO or EC. In earlier trials we had observed such sensory reduction to affect PPG frequencies (Perlitz, Cotuk, Schiepek, et al., 2004), which we considered in keeping with such effects on EEG complexity (Müller et al., 2003). Whether and how this was influenced by a significant main effect of examiner for LF and IM amplitudes (LF: *p* < 0.01; IM: *p* < 0.05) needs further study with emphasis on coordination and synchronization between examiner and participant.

### 4.6 ECG—HRV

So far, heterogeneous findings have been reported for time‐ or frequency‐domain measures (Rechberger et al., 2019). In this study, we included standard HRV measures before, during and following CVH. In a linear effects model, the mean HR decreased significantly from section 1 to CVH, and 2 to CVH as well as from section 2 to 4. Furthermore, the mean HR was higher during T1 compared to T0 measurements. This may be related to a general adaptation during enhanced relaxation. We did not observe any effects within RMSSD, LogLFpower, LogIMpower and LogHFpower. This is in contrast to studies that found an increase of, for example, HF‐HRV parameters during and directly following OMT treatment in healthy non‐symptomatic adults which suggested a modulation of parasympathetic outflow (Arienti et al., 2020; Cerritelli et al., 2020; Henley et al., 2008; Rechberger et al., 2019). This also contrasts clearly with dynamic changes we have observed in PPG data. However, the interpretation of previous studies was often limited, for example by use of the poorly consistent LF/HF ratio (see above) (Berntson et al., 1997). Another problem pertains to the existence of additional rhythms possibly indexing the CRI, which cannot be explained using conventional frequency domain measures such as the LF/HF ratio (McPartland & Mein, 1997; Rasmussen & Meulengracht, 2021). We therefore suggest that standard HRV measures fail to capture the higher complexity of physiological changes. This further supports the necessity to establish a “second generation model” for HRV research which respects also its nonstationary and nonlinear dynamics (Perlitz, Cotuk, Lambertz, et al., 2004; Schwerdtfeger et al., 2020). Importantly, the comparison of ECG‐based HRV measures and physiological reactions in PPG needs to be interpreted with care as HRV is strongly linked to respiration and BP waves and frequency responses of HRV were not presented.

### 4.7 Limitations

There are several limitations to generalization of our findings. First, the purpose of our study was to further our understanding of IM band physiology with respect to CVH intervention and internal control conditions. However, in future studies, a between‐subjects control (e.g., sham) condition should be included to be able to compare effects of CVH as to its specificity. Second, we applied analyses of time‐ and frequency‐domain as well as amplitude measures of different physiological signals. Further analyses should include dynamic interactions of these physiological systems. This should also include physiology of the osteopathic examiner to fully understand the biodynamic interplay within and between examiner and patient. Third, adding another frequency band (IM band) still does not resolve the problem of rigid frequency bands. However, it offers a better fit within the much‐disputed LF range (Schwerdtfeger et al., 2020). Fourthly, in our previous analysis of the data (preprint: Pelz et al., 2023) based on 31 measurements, we reported a ratio of 77% participants exhibiting slowed LF PPG oscillations and 23% remaining in IM mode. The current analyses with extended sample (55 measurements), however, resulted in an approximate 50/50 ratio. Fifthly, even though the dichotomous LF‐HF concept has originally been established for the analysis of ECG‐based HRV and BP data (Camm et al., 1996), we transferred this concept to the analysis of PPG data. This seems justified in light of earlier findings showing 0.15 Hz activity in parallel in time series of PPG and ECG derived HRV (see Figure 3 and six in (Perlitz, Cotuk, Schiepek, et al., 2004); see Figure 1d,f in Perlitz, Lambertz, Cotuk, et al., 2004). Lastly, our analyses are based on MFHA and TFD which have inherent limitations such as suppressing frequencies of minor amplitude and some frequency uncertainty particularly in lower frequencies.

## 5 CONCLUSIONS

A cranial osteopathic intervention (CVH), applied in the supine position, was associated with changes in autonomic output to the SP and respiration in 38 healthy adults. Contrary to explaining the observed rhythms during CVH as Traube‐Hering waves (e.g., Nelson et al., 2006), we propose our current data and previously reported findings in the literature support a dynamic model of three frequency bands including IM physiology. We report rhythmic responses in the LF band to CVH in forehead SP in approximately half of the participants, which were comparable with adaptive responses reported for IM activity in autogenic relaxation. In the other group of similar size, participants responded to CVH with activation of activity in the lower LF range, which we interpret to represent subharmonics of the primary IM band or activation of baroreflex pathways. Over the course of two separate recordings, there was a general increase of ANS related frequencies in SP approaching frequencies essential for the physiology of the IM band with central pacemaker activity in the brainstem at 0.15 Hz (± 0.03 Hz) in the IMR group, activating also interoceptive networks. Presence of lower and upper harmonic frequencies generating integer number couplings in respiration and PPG appear plausible but warrant further analysis (Lambertz & Langhorst, 1998; Perlitz, Cotuk, Lambertz, et al., 2004; Perlitz, Lambertz, Cotuk, et al., 2004). Hopefully, our current findings help overcome the “lack of information available to describe the potential link between changes in SP and similar changes in the blood circulation in deeper tissues” stated for OMT research (p. 914; Zegarra‐Parodi et al., 2014) since IM band activity has been found to take effect on all stages of the organism.

## AUTHOR CONTRIBUTIONS

Holger Pelz and Volker Perlitz conceived the idea for the study. Holger Pelz, Volker Perlitz, Micha Keller, Gero Müller, and Klaus Mathiak contributed to the design and planning of the research. Holger Pelz and Johannes Mayer were involved in data collection. Gero Müller and Stefan Borik pre‐processed the data Volker Perlitz, Gero Müller, Micha Keller and Stefan Borik analyzed the data. Holger Pelz coordinated funding of the project. All authors edited and approved the final version of the manuscript.

## CONFLICT OF INTEREST STATEMENT

The authors declare no competing interests.

## ETHICS STATEMENT

Subjects provided written, informed consent to the current protocol that was approved by the Institutional Review Board of the state of Lower‐Saxony/Germany (EK vote from 04/03/2017) and conducted in accordance with the Declaration of Helsinki.

## Data Availability

The datasets used and/or analyzed during the current study are available from the corresponding author on reasonable request.
